# Humoral responses to SARS-CoV-2 vaccination in allogeneic hematopoietic cell transplantation patients resemble that of healthy individuals

**DOI:** 10.3389/fimmu.2026.1829281

**Published:** 2026-07-01

**Authors:** Claus-Philipp Maier, Beate Preuss, Kristina Reuss, Jonas Welter, Ann-Christin Pecher, Wichard Vogel, Christoph Faul, Wolfgang Bethge, Reinhild Klein

**Affiliations:** 1 Department of Hematology, Oncology, Clinical Immunology, and Rheumatology, Medical Clinic, University of Tuebingen, Tuebingen, Germany; 2 Department of Hematology, Oncology, Gastroenterology, Nephrology, Rheumatology, Children’s Hospital, University of Tuebingen, Tuebingen, Germany

**Keywords:** allogeneic hematopoietic cell transplantation (alloHCT), anti-nucleocapsid antibodies, anti-spike-1 antibodies, anti-spike-RBD antibodies, SARS-CoV-2, vaccine immunogenicity

## Abstract

**Introduction:**

Allogeneic hematopoietic cell transplantation (alloHCT) recipients are at increased risk of severe COVID-19. Although vaccination is strongly recommended, immune responses may vary and can be affected by clinical and treatment-related factors.

**Methods:**

In this longitudinal, single-center study, we analyzed IgG, IgM, and IgA antibody kinetics to severe acute respiratory syndrome-Corona virus-2 (SARS-CoV-2) spike-1, receptor binding domain (RBD), and nucleocapsid proteins in 354 alloHCT patients and 647 healthy controls by ELISA. A total of 1,364 serum samples were collected before and after one, two, and three doses of mRNA, adenovirus vector, and/or protein-based vaccines. Clinical parameters, including age, conditioning regimen, immunosuppressive therapy, graft-versus-host disease (GVHD), donor type, time since transplantation, diagnosis, and laboratory parameters were evaluated for their influence on antibody responses.

**Results:**

Anti-spike-1 IgG responses increased progressively: 27% of patients versus 28% of controls seroconverted after the first vaccination, 65% versus 59% after the second, and 93% versus 98% after the third. After three doses, no significant differences in IgG prevalence or reactivity remained between groups. IgM and IgA responses were infrequent. Multivariate analysis identified diagnosis, shorter interval between transplantation or vaccination and antibody testing, and lower lymphocyte count as negative predictors after the first vaccination. No negative predictors were detected after the third dose. Antibody levels remained stable for at least six months following the third vaccination. Breakthrough infections occurred but were mild.

**Conclusions:**

AlloHCT recipients achieve humoral immunity comparable to healthy individuals after three vaccine doses, supporting efficacy and safety of repeated SARS-CoV-2 vaccination in this vulnerable population.

## 1 Introduction

Patients undergoing allogeneic hematopoietic cell transplantation (alloHCT) are at an increased risk of severe SARS-CoV-2 infection due to immunosuppression and comorbidities (1, 2). Professional societies such as the European Society for Blood and Marrow Transplantation (EBMT), the American Society for Transplantation and Cellular Therapy (ASTCT), and the American Society of Hematology (ASH) recommend vaccination as early as three months post-transplant (2, 3). However, vaccine-induced immune responses vary depending on immunosuppressive therapy, lymphopenia, hypogammaglobulinemia (especially IgA deficiency), and age (> 60–65 years), whereas conditioning intensity, chronic graft-versus-host disease (GVHD), donor type, and transplantation type (i.e. identical, mismatched or haploidentical), appear to have a limited impact (3–8).

Five vaccines were approved in the European Union during the 2020–2022 pandemic: mRNA-based vaccines (BNT162b2 [Pfizer/Biontech]; mRNA-1273 [Moderna]), non-replicating adenoviral vector vaccines (ChAdOx1-S [AstraZeneca], Ad26.COV2-S [Johnson&Johnson/Jansen]), and the recombinant protein vaccine NVX-CoV2373 (Novavax) (9). mRNA vaccines have been used most widely in alloHCT recipients and are recommended by the EBMT (9).

SARS-CoV2 contains four essential structural proteins that are critical for the virus’s assembly and replication (10). The spike protein, consisting of S1 and S2 subunits, is located on the viral surface. The S1 subunit contains the receptor-binding domain (RBD), which interacts with the cellular receptor angiotensin-converting enzyme 2 (ACE2) and mediates viral entry into host cells. The nucleocapsid protein is located within the virion and surrounds the viral RNA genome. In addition, SARS-CoV-2 contains the membrane (M) protein, the major component of the viral envelope, and the envelope (E) protein, which contributes to virion assembly, release and infectivity. The spike and nucleocapsid proteins are of particular importance for clinical diagnostics, assessment of immunity and vaccine development. Antibodies against the spike protein are induced by both infection and vaccination, whereas antibodies against the nucleocapsid protein are generally indicative of natural infection, as this antigen is not included in currently approved COVID-19 vaccines (9, 10). Consequently, serological assays targeting the spike-1 protein, the RBD, and the nucleocapsid protein have been developed. Antibodies directed against the RBD correlate strongly with neutralizing activity because they can block viral entry into host cells. Furthermore, the RBD is poorly conserved among human corona viruses resulting in high specificity for infection with SARS-CoV-2. In contrast, spike-1 antibodies may show limited cross-reactivity with other corona viruses. However, such cross-reactivity is considered unlikely to comprise affect diagnostic accuracy because infections with these viruses are relatively uncommon in humans (11).

Following two vaccine doses, seroconversion rates in alloHCT recipients exceed 75% but this figure remains lower than that observed in healthy individuals (3, 12, 13). A third dose significantly improves responses without causing major side effects (9, 13–16). Vaccination is generally well tolerated, with GVHD exacerbations occurring only rarely (6, 17). However, there are only a few longitudinal studies comparing immune reactivity in healthy individuals and alloHCT-recipients after three vaccinations. Most available studies have included only small groups of individuals, and have primarily focused on responses following the first two vaccine doses (3, 18).

In the present study, we, therefore, investigated the longitudinal development of IgG-, IgA-, and IgM antibodies directed against the spike-1, the RBD- and the nucleocapsid proteins in a large group of alloHCT recipients and healthy individuals during a three-dose vaccination regimen. Furthermore, we evaluated clinical and laboratory parameters associated with antibody induction to gain further insights into factors influencing vaccine-induced humoral immunity.

## 2 Patients

Between March 1, 2021, and December 31, 2022, sera from 354 alloHCT recipients were included in the study. The underlying diagnoses were as follows: acute leukemia (n=196), chronic leukemia (n=37), myelodysplastic syndrome (MDS, n=46), myeloproliferative neoplasms (MPN, n=27), B-cell non-Hodgkin lymphoma (B-NHL, n=11), chronic myelomonocytic leukemia (CMML, n=7), T-NHL (n=8), other malignant hematological disorders (n=7), and non-malignant hematological/other disorders (n=8). A total of 1,054 serum samples were analyzed. Detailed baseline characteristics, including leukocyte and lymphocyte counts and serum immunoglobulin (IgG, IgA, IgM) levels at the first serological assessment, are summarized in **Supplementary Table 1**; further diagnostic and demographic information can be found in **Supplementary Table 2**. The severity of chronic GVHD was scored according to Jagasia et al. (19).

Patients with less than six months of follow-up were excluded from the study. Additionally, patients receiving B-cell-depleting antibodies were excluded since this type of therapy may falsify the data by preventing antibody production.

As controls, sera from 647 healthy medical staff members (not matched) were analyzed obtained before and/or after vaccination as part of the clinical routine.

All samples were stored at −20 °C.

The study was conducted according to the Declaration of Helsinki and was approved by the local ethics committee (no. 0704-BO_retro).

## 3 Methods

### 3.1 ELISA

Antibodies against spike-1, spike RBD, and nucleocapsid proteins were measured using an in-house ELISA based on a previously described autoantibody detection method (20). Optimal antigen and serum concentrations were determined prior to the study.

Nunc™ MicroWell™ 96-well plates (Thermo Fisher Scientific) were coated with Spike S1-His (0.1 µg/ml; SinoBiological, Beijing, China), Spike RBD-His (0.3 µg/ml; GenScript, Piscataway, NJ, USA) or Nucleocapsid-His recombinant proteins (0.1 µg/ml, SinoBiological). Patient sera were diluted 1:500 for IgG and IgM, and 1:100 for IgA antibody detection. Peroxidase-conjugated anti-human IgG, IgM, and IgA antibodies were applied at dilutions 1:3,000, 1:2,000, and 1:667, respectively (Dianova, Hamburg, Germany). Ortho-phenylenediamine (Sigma-Aldrich/Merck, Darmstadt, Germany) was used as substrate.

Initially, antibody reactivity was expressed as binding antibody units per milliliter (BAU/ml) using the WHO working standard 21/234. Positive and negative samples were defined based on this standard. Once this standard became unavailable, arbitrary units (AU) were used: the optical density (OD) of a positive control was divided by the OD of a negative control, multiplied by the OD of the test sample and scaled by 100. The same positive controls were used on each assay plate for standardization.

### 3.2 Statistics

Statistical analyses were performed using GraphPad Prism 9.2.0 and IBM SPSS 28. Nonparametric tests were applied: Mann–Whitney U test for unpaired comparisons, Spearman correlation for nonparametric correlations [r>0.5 indicates a weak, r=0.5–0.7 a moderate, r >0.7 a high correlation (21)], and Fisher’s exact test for comparison of antibody prevalence between groups.

The predictive effects of selected clinical characteristics and laboratory parameters on the antibody response to vaccination were assessed using univariate and multivariate logistic regression.

P-values < 0.05 were considered statistically significant.

## 4 Results

### 4.1 Patient characteristics

The median age of the 354 alloHCT recipients was 60 years (range 18–93 years) (**Supplementary Table 1B**). Of these patients, 345 had undergone alloHCT before study enrollment (median interval between alloHCT and first serum analysis: 43 months, range 0.7-364.7 months), while the remaining nine patients underwent alloHCT during the observation period. Related alloHCT was performed in 118 patients (identical: n=93; haploidentical: n=25), and unrelated alloHCT in 227 patients (identical: n=177; mismatched: n=50) (**Supplementary Table 1B**).

The median interval between transplantation and the first vaccination was 66.3 months (range, 7.7 months prior to HSCT through 354.3 months post HSCT; **Supplementary Table 1A**). The time between alloHCT and the first vaccination was between 0 and 6 months for twenty-two samples.

The median time between the first vaccination and antibody measurement was 17 days (range 1-138.6 days). The first vaccination was performed using the mRNA vaccines BNT162b2 and mRNA-1273 in 76% and 5% of patients, respectively, and the vector vaccines ChAdOx1-S and Ad26.COV2-S in 15% and 1% of patients, respectively.

Reduced-intensity (RIC) and myeloablative conditioning (MAC) were equally represented (177 patients, each). Immunosuppressive treatment following alloHCT is summarized in **Supplementary Table 1A**. Of the patients who underwent the first serological analysis, 54 were still receiving immunosuppressive therapy, while 300 had stopped treatment.

Chronic graft-versus-host disease (GVHD) was classified as none, mild, moderate, and severe (19). At the time of the first vaccination, 242 patients had no GVHD (group 1), 72 patients had mild (group 2), 28 moderate (group 3), and 12 patients severe GVHD (group 4) (**Supplementary Table 1A**).

During the observation period, 16 patients (acute lymphatic leukemia [ALL] n=10, MPN n=1, MDS n=1, B non-Hodgkin Lymphoma [NHL] n=1, chronic myeloid leukemia [CML] n=1, blastic plasmacytoid dendritic cell neoplasm [BPDCN] n=1, and severe aplastic anemia [SAA] n=1) received antibodies against SARS-CoV-2, either for prophylaxis or treatment of SARS-CoV-2 infection (Tixagevimab/Cilgavimab [Evusheld^®^] n=8, Sotrovimab [Xevudy^®^] n=9, and Casirivimab/Imdevimab [Ronapreve^®^] n=7).

A total of 50 patients became infected with SARS-CoV-2 during the observation period, primarily before or between the first and second vaccinations (**Supplementary Table 3**). None of the patients died because of SARS-CoV-2 infection.

The median age of the 647 healthy controls was 39 years (range 18-80); of these, 472 were female, and 175 were male. Of the controls, 54 were infected with SARS-CoV-2 during the observation period; 35 (65%) of these cases occurred before the first vaccination (**Supplementary Table 3**).

The prevalence of SARS-CoV-2 infection was significantly higher before and after first vaccination among patients with hematological disorders (16% and 14%) than in healthy controls (8% and 3%) (p<0.01 and p<0.05, respectively). Conversely, after the third vaccination, SARS-CoV-2 infection occurred more frequently in healthy individuals (10%) than in patients with hematological disorders (3%; p<0.001) (**Supplementary Table 3**).

### 4.2 Antibody responses after vaccination

**Table 1** summarizes the prevalence and kinetics of IgG, IgM, and IgA antibodies against spike-1, spike-RBD, and the nucleocapsid following vaccination (independent of vaccine type), in patients with hematological disorders after alloHCT and in healthy controls.

**Table 1:** Prevalence of IgG-, IgM- and IgA-antibodies to different antigens of SARS-CoV-2 in patients after alloHCT and in healthy controls in relation to number of vaccinations.

Time point	Healthy individuals	Patients	Healthy individuals	Patients	Healthy individuals	Patients
	IgG antibodies		IgM antibodies		IgA antibodies	
	Number positive/number tested (%)					
Antibodies to spike-1						
Before 1^st^ vaccination	42/449 (9)^a)^	20/145 (14) ^a)^	5/435 (1)	2/124 (2)	0/449 (0)	2/145 (1)
After 1^st^ vaccination	10/36 (28) ^**)^	28/103 (27) ^*)^	2/31 (6)	2/69 (3)	0/36 (0)	2/103 (2)
After 2^nd^ vaccination	79/135 (59) ^****)^	170/262 (65) ^****)^	6/98 (6) ^**)^	3/122 (2)	1/135 (1)	2/262 (1)
After 3^rd^ vaccination	109/111 (98) ^****)^	114/123 (93) ^****)^	n.t.	n.t.	4/111 (4) ^**)^	6/122 (5)
Antibodies to spike-RBD						
Before 1^st^ vaccination	66/449 (15)	8/124 (6)	1/429 (0.2)	1/124 (1)	1/448 (0.2)	2/145 (1)
After 1^st^ vaccination	7/36 (19)	24/103 (23) ^****)^	0/31 (0)	2/69 (3)	0/36 (0)	5/103 (5)
After 2^nd^ vaccination	52/135 (26) ^****)^	130/262 (50) ^****)^	3/98 (3) ^*)^	0/122 (0)	3/135 (2) ^*)^	8/262 (3)
After 3^rd^ vaccination	103/111 (93) ^****)^	106/123 (86) ^****)^	n.t.	n.t.	12/111 (11) ^****)^	19/122 (16) ^****)^
Antibodies to nucleocapsid						
Before 1^st^ vaccination	57/448 (13) ^a)^	21/145 (14) ^a)^	20/428 (5)	7/124 (6)	6/448 (1)	2/145 (1)
After 1^st^ vaccination	10/36 (28) ^*)^	17/103 (17)	2/31 (6)	4/69 (6)	2/36 (6)	0/103 (0)
After 2^nd^ vaccination	29/135 (21) ^*)^	37/262 (14)	10/98 (10) ^*)^	6/122 (5)	2/135 (1)	5/262 (2)
After 3^rd^ vaccination	27/111 (24) ^*)^	18/122 (15)	n.t.	n.t.	3/111 (3)	3/122 (2)

^a)^ 24 of the patients and 35 of the healthy individuals had suffered from COVID19 infection as verified by PCR.

significance levels as compared to the time before vaccination: Significance levels: *p < 0.05; **p <0.01; ***p < 0.001; ****p < 0.0001.

There were no significant differences between patients and healthy individuals at any time point except for anti-spike-RBD IgG antibodies after second vaccination (50% vs 26%, p=0.04).

Serum samples from patients who received anti-SARS-CoV-2 antibodies prophylactically and/or therapeutically were excluded from the analysis.

n.t.: not tested; due to the low prevalence of anti-SARS-CoV-2 antibodies of the IgM type, the antibodies were not any more analyzed at time points beyond 2^nd^ vaccination

Prior to the first vaccination, 6-14% of patients and 9-15% of healthy individuals already exhibited IgG antibodies against the spike-1, the spike-RBD, and the nucleocapsid proteins. All these antibody positive patients and 35 of the healthy individuals had a confirmed SARS-CoV-2 infection via PCR testing and exhibited clinical symptoms; the remaining seropositive individuals may have experienced asymptomatic infections.

In patients with hematological disorders and healthy individuals, the prevalence of anti-spike-1 IgG antibodies increased significantly after the first vaccination (**Table 1**), while anti-spike-RBD IgG antibodies increased only in the patients, although most remained seronegative at this time. Following the third vaccination, 93% of patients and 98% of controls developed anti-spike-1 IgG antibodies, while anti-spike-RBD IgG antibodies were present in 86% and 93%, respectively. As expected, vaccination had little effect on nucleocapsid-specific IgG prevalence. The increase in nucleocapsid IgG seropositivity observed in healthy individuals likely reflects undetected and/or clinically inapparent SARS-CoV-2 infections in the context of reduced PCR testing after the third vaccination in 2022.

Throughout the observation period, no significant differences in anti-SARS-CoV-2 antibody prevalence were detected between patients and controls, except for anti-spike-RBD antibodies after the second vaccination (50% vs. 26%, p=0.04) (**Table 1**).

IgM and IgA antibodies against SARS-CoV-2 antigens were rarely detected either before or after vaccination (**Table 1**). Consequently, subsequent analyses focused on IgG responses.

**Figure 1:**
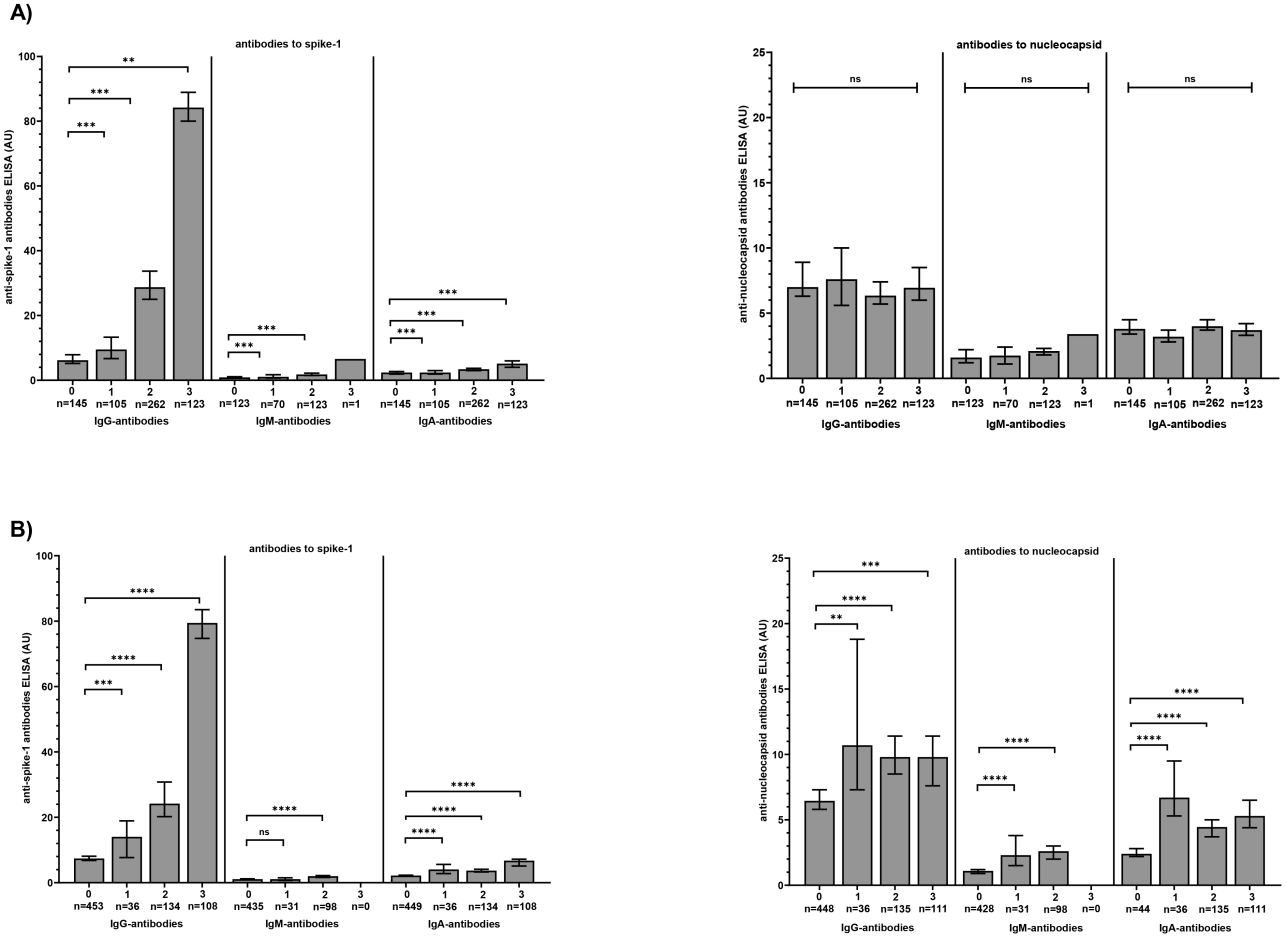
Reactivity of IgG-, IgM- and IgA-antibodies to the spike-1 protein and nucleocapsid of SARS-CoV-2 before and after vaccination in patients with hematological disorders **(A)** and healthy individuals **(B)**. Time points: 0 = before vaccination; 1 = after 1^st^ vaccination; 2 = after 2^nd^ vaccination; 3 = after 3^rd^ vaccination. AU: arbitrary units; ns: not significant. Median ± 95% CI is given. Timepoint 3: Due to the low number of patients developing IgM-antibodies, these antibodies were analyzed only until time point 2. Significance levels: * p < 0.05; ** p < 0.01; *** p < 0.001; **** p < 0.0001 There was no significant difference between patients and healthy individuals at any timepoint. Serum samples from patients who received anti-SARS-CoV-2 antibodies were excluded from the analysis.

A significant increase in anti-spike-1 IgG reactivity was observed in both groups already after the first vaccination (**Figures 1A**, **B**), and similar findings were observed for anti-spike-RBD IgG (data not shown). Notably, no significant differences in IgG reactivity were observed between patients and controls at any vaccination time point. Nucleocapsid-specific IgG reactivity increased significantly only in the control group, consistent with ongoing SARS-CoV-2 exposure. However, the values remained close to the cut-off thresholds: 20.0 AU for IgG and IgA and 6.0 AU for IgM.

Comparing the reactivity of IgG anti-spike-1 antibodies in patients with and without a prior SARS-CoV-2 infection before the first vaccination revealed that, as expected, reactivity was significantly higher in patients with a prior infection, both before and after the first vaccination. However, no significant differences were observed between the two groups after the second and third vaccinations (see **Supplementary Figure 1**).

### 4.3 Influence of sex and age

**Figure 2:**
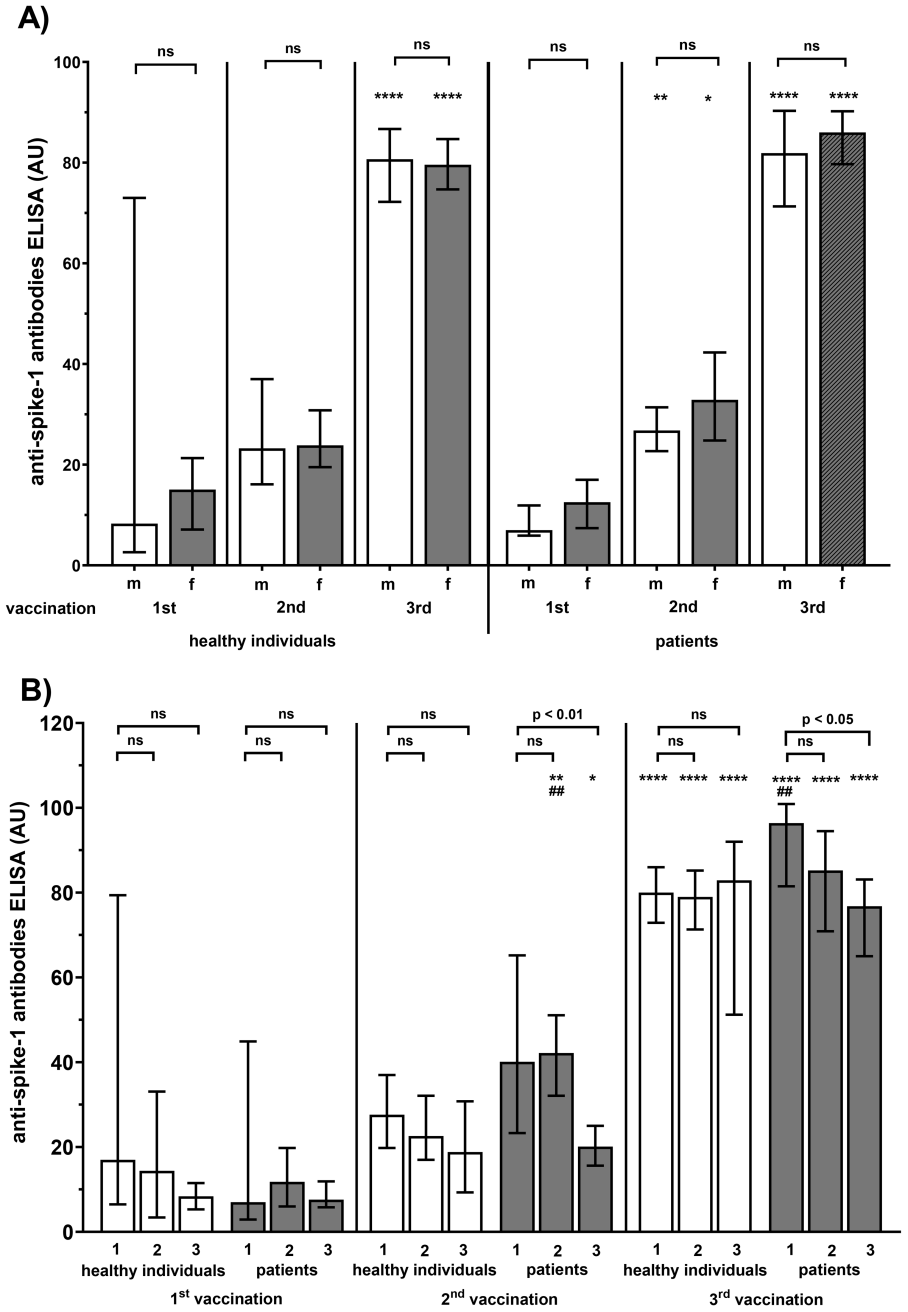
Sex- **(A)** and age- **(B)** relation of anti-spike-1 IgG antibody reactivity after vaccination in healthy individuals and patients with hematological disorders. M=male, f=female; age groups: 1 = < 40 years, 2 = 40–60 years, 3 = > 60 years. Significance levels as compared to 1^st^ vaccination of the same group: *p < 0.05; **p < 0.01; ***p < 0.001; ****p < 0.0001. Significance levels as compared to healthy individuals: ^##^p < 0.01. AU, arbitrary units; ns, not significant. Median ± 95% CI is given. Serum samples from patients who received anti-SARS-CoV-2 antibodies were excluded from the analysis.

No sex-related differences in antibody induction were observed between patients or controls after any vaccination (**Figure 2A**). In patients, significant increases in anti-spike-1 IgG reactivity were observed in both sexes after the second vaccination, whereas in healthy individuals this was only observed after the third dose.

When stratified by age at time of the start of the study (< 40 years, 40–60 years, > 60 years), patients over 60 years of age displayed significantly lower IgG antibody levels than younger patients after the second and third vaccinations (**Figure 2B**). This age effect was not observed in healthy controls. Patients over 40 years of age showed significant increases in antibody reactivity after the second vaccination compared to the first, while controls required the third vaccination to achieve comparable increases.

### 4.4 Time after vaccination

No correlation was observed between spike-1 IgG antibody reactivity and time since vaccination for any of the three vaccinations, in either healthy individuals (Spearman correlation coefficient r: 0.04-0.22) or patients (r: -0.398-0.44) (**Supplementary Figure 2**).

**Figure 3:**
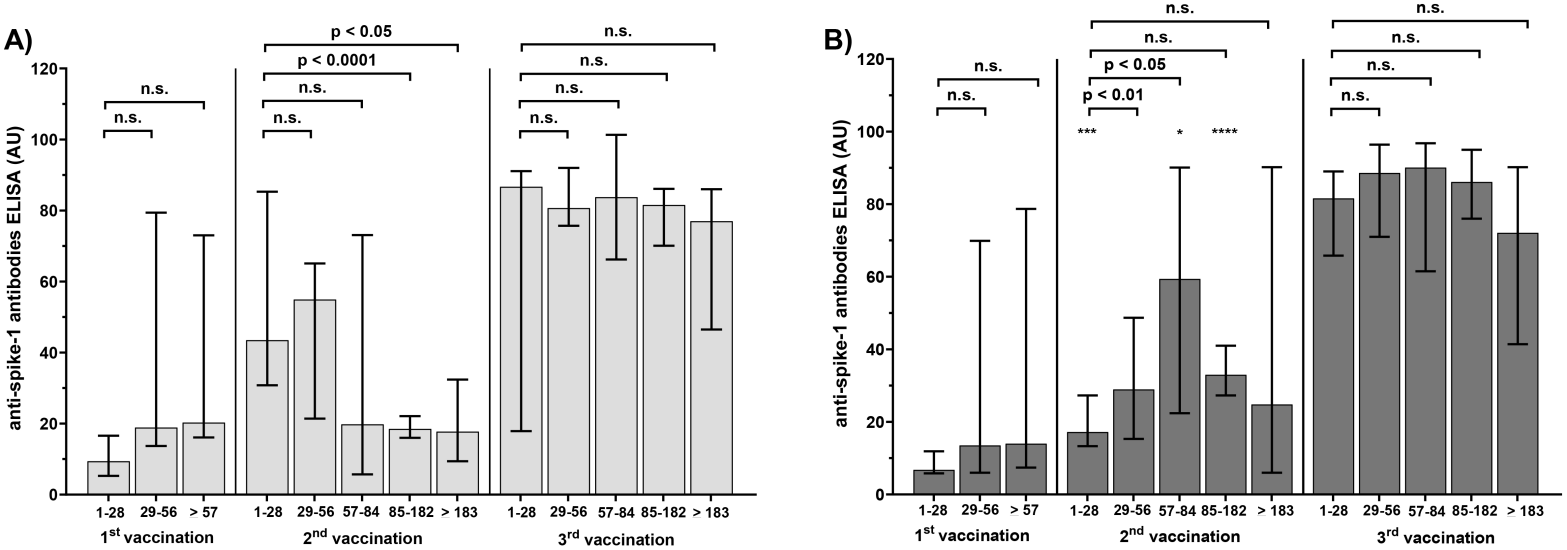
Correlation between time after vaccinations (in days) and IgG-anti-spike-1 antibody production in healthy individuals **(A)** and in patients with hematological disorders after HCT **(B)** in relation to the number of vaccinations. Significant as compared to antibody reactivity in healthy individuals after the same time of vaccination *p < 0.05; ***p < 0.001; ****p < 0.0001; After first and third vaccination, there were no significant differences between patients and healthy individuals. AU: arbitrary units; n.s.: not significant. Median ± 95% CI is given.

Stratification by interval (for 1^st^ vaccination: days 1-28, days 29-56, ≥ 57 days after vaccination; for 2^nd^ and 3^rd^ vaccinations: days 1-28, days 29-56, days 57-84, days 85-182, ≥183 days after vaccination) revealed a statistically not significant increase in antibody reactivity 29 days after the first vaccination in patients and controls (**Figures 3A**, **B**). Following the second vaccination, antibody reactivity increased between days 29–56 in controls, but declined thereafter. In contrast, patients exhibited significant increases beyond day 29 (**Figure 3B**). Following the third vaccination, an increase was observed as early as days 1–28 in both, patients and healthy controls, with antibody reactivity remaining nearly unchanged for over six months.

Following the first vaccination, there was no difference in antibody responses observed between patients and controls at any time point (**Figures 3A**, **B**). Following the second vaccination, patients exhibited a delayed increase compared to controls, which however lasted for a longer period (days 57-182). No group differences were seen after the third vaccination. Additionally, there was no significant difference in antibody prevalence between healthy controls and alloHCT patients after three vaccinations.

### 4.5 Vaccine type

After the first dose, seroconversion ranged from 20-30% with mRNA vaccines (**Supplementary Table 4**). None of the 15 patients receiving ChAdOx1-S seroconverted.

After two doses, seroconversion reached 58-80% depending on the vaccine used. After three doses, > 81% seroconverted. Notably, all patients who received mixed vaccination regimens eventually developed anti-spike-1 IgG antibodies after the third dose. Only three patients received mRNA-1273 for all three vaccinations, and all three had seroconverted by the time they received the third dose.

As differences between vaccine types were small, subsequent analysis did not stratify by vaccine.

### 4.6 Underlying disease and transplant characteristics

**Figure 4:**
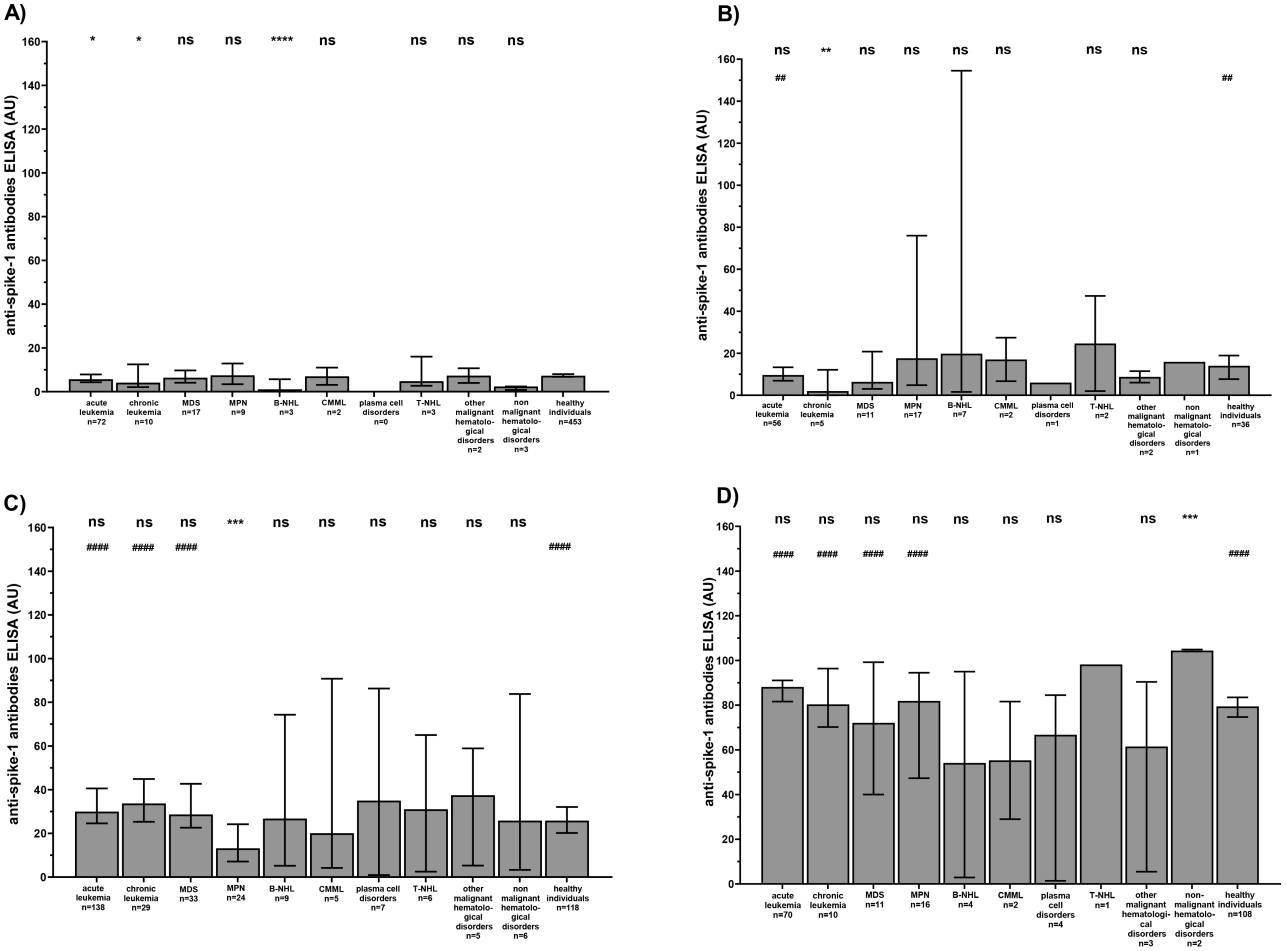
Reactivity of IgG-anti-spike-1-antibodies in patients with different hematological disorders and healthy controls in relation to the number of vaccinations **(A)** before vaccination, **(B)** after 1^st^ vaccination, **(C)** after 2^nd^ vaccination, **(D)** after 3^rd^ vaccination. AU: arbitrary units; ns: not significant. Median ± 95% CI is given. Significance levels as compared to healthy individuals: n.s. = not significant; *p < 0.05; **p < 0.01; ***p < 0.001; Significance levels as compared to baseline reactivity before first vaccination: ^#^p < 0.05; ^##^p < 0.01; ^###^p < 0.001; ^####^p < 0.0001. MDS, myelodysplastic syndrome; MPN, myeloproliferative neoplasms; NHL, Non-Hodgkin lymphoma; CMML, chronic myelomonocytic leukemia.

Anti-spike-1 IgG reactivity increased significantly in healthy individuals and patients with acute leukemia after the first, in patients with chronic leukemia and MDS after the second, and in patients with MPN after the third vaccination (**Figure 4**). In the other groups, the number of patients was too low to calculate statistical significance. Baseline antibody titers were lower in patients with acute and chronic leukemia than in the control group.

Additionally, the prevalence of spike-1 IgG antibodies increased significantly in all patient groups except for patients with MPN and in healthy controls after the second vaccination. A significant increase was already observed after the first vaccination in patients with acute leukemia and in healthy individuals. Interestingly, the prevalence of anti-spike-1 antibodies did not differ between healthy controls and patients at any time point, irrespective of the underlying disease (**Supplementary Table 5**).

The apparently higher responses in patients with non-malignant hematological disorders are likely due to the small sample size.

Donor type had no significant impact (**Supplementary Figure 3**). However, patients with unrelated identical donors showed significant antibody increases after the second vaccination, whereas those with mismatched unrelated or related identical transplants only responded after the third vaccination. Haploidentical transplant recipients showed increased antibody levels after the second and third vaccinations, though the differences from baseline did not reach statistical significance.

MAC recipients showed stronger responses after the second vaccination than RIC recipients but these differences disappeared after the third dose (**Supplementary Figure 4A**). Notably, total serum IgG levels were significantly lower in the MAC than in the RIC group after third vaccination while there was no difference in leukocyte and lymphocyte counts between the two groups (**Supplementary Figures 4B–D**).

### 4.7 Immunosuppression and GVHD

Ongoing immunosuppressive therapy was associated with reduced anti-spike-1 IgG antibody responses after the second vaccination but not after the third (**Supplementary Figure S5A**). There was no association with a distinct therapeutic regime (MMF vs Tacrolimus vs CSA).

Mild GVHD was associated with higher antibody reactivity after the first vaccination. However, no differences between GVHD groups remained after the third vaccination (**Supplementary Figure 5B**). Notably, a significant increase in anti–spike-1 IgG reactivity was already evident after the second vaccination in patients without GVHD. In contrast, this increase only became apparent in patients with mild, moderate, and severe GVHD only after the third vaccination (**Supplementary Figure 5B**).

### 4.8 Timing of vaccination after alloHCT

**Figure 5:**
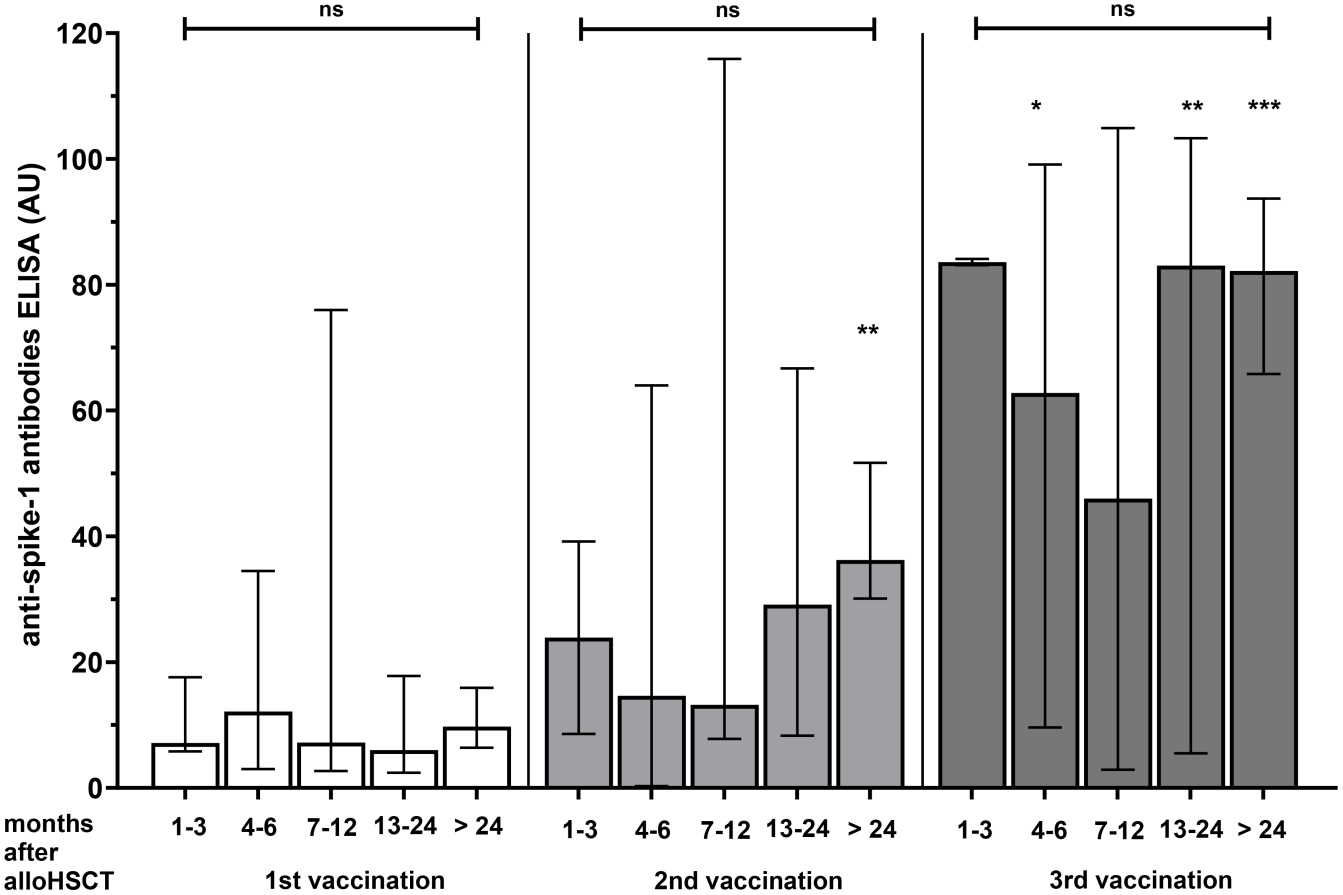
Relation between time after alloHCT (months) and induction of anti-spike-1 IgG antibodies after the three vaccinations. No significant differences were observed comparing patients after short follow up after HCT and those followed for several months after HCT. Significant as compared to antibody reactivity after first vaccination: *p < 0.05; **p < 0.01; ***p < 0.001. AU, arbitrary units; ns, not significant. Median ± 95% CI is given.

No strong correlation was observed between the timing of vaccination after alloHCT and antibody responses (**Figure 5**). When patients were stratified into two groups, i.e. those vaccinated within 24 months after HCT (1^st^ vacc. n=46, 2^nd^ vacc. n=27, 3^rd^ vacc. n=18) and those vaccinated more than 24 months after HCT (1^st^ vacc. n=54, 2^nd^ vacc. n=84, 3^rd^ vacc. n=27), a significant difference was only observed after the second vaccination (first vaccination: median: 7.4 AU vs. 9.8 AU, not significant; second vaccination: median: 17.2 AU vs. 36.3 AU, p < 0.05; third vaccination: median: 82.3 AU vs. 82.2 AU, not significant).

Correlation analyses revealed no association between time since alloHCT and anti-spike-1 IgG responses (r= 0.13-0.15; **Supplementary Figure 6**). In a few patients, vaccination was performed as early as one month post alloHCT (data not shown); yet, antibody induction was comparable across time intervals.

### 4.9 Laboratory parameters and antibody response

Several reports indicate that lymphopenia or hypogammaglobulinemia of the IgG- or IgA-type may negatively impact the induction of anti-spike-1 IgG antibodies after vaccination in alloHCT patients (22, 23). In the present study, correlations between antibody levels, leukocyte counts and lymphocyte counts, as well as immunoglobulin levels were weak (r= -0.22 – 0.29; **Supplementary Figure 7**).

### 4.10 Clinical and laboratory parameters in non-responder vs. responder after third vaccination

The following clinical and laboratory parameters were analyzed in non-responders (n=9) vs. responders (n=114) after the third vaccination: gender, age, intensity of conditioning, immunosuppressive therapy, GVHD severity, time from HCT to blood sampling, time from vaccination to blood sampling, and levels of leukocytes, lymphocytes, and serum IgG, IgA, and IgM immunoglobulins. Significant differences were observed in lymphocyte counts (non-responder: mean 1.4 ± 0.8 G/l median 1.1 G/l; responder: 2.0 ± 1.1 G/l, median 1.8 G/l; p<0.05), serum IgA levels (36 ± 21 mg/dl, median 24 mg/dl, and 146 ± 117 mg/dl, median 113 mg/dl, respectively; p<0.05), and serum IgM levels (33 ± 27 mg/dl, median 21 mg/dl, and 98 ± 65 mg/dl, median 83 mg/dl, respectively; p<0.05) (data not shown). However, the low number of antibody-negative patients may have hampered the statistical analysis.

### 4.11 Monoclonal antibody therapy

Sixteen patients received monoclonal antibody therapy during follow-up (**Supplementary Figure 8**). This treatment increased anti-spike-1 and, to a lesser extent, anti-spike-RBD IgG reactivity, without affecting nucleocapsid antibodies (**Supplementary Figures 8a–h**). In some cases, it was not possible to determine whether the antibodies originated from vaccination or therapy (**Supplementary Figures 8i–k**). The lack of effect in one patient (**Supplementary Figure 8l**) was probably due to delayed sampling after therapy (eight months). In some cases, the antibodies were administered concurrently with a SARS-CoV-2 infection (**Supplementary Figures 8m, n**). One patient who was already showing strong vaccine-induced reactivity did not demonstrate an increased antibody response following prophylactic treatment (**Supplementary Figure 8o**). Five patients developed PCR-confirmed mild SARS-CoV-2 infection despite prior vaccination and/or antibody therapy (**Supplementary Figures 8e, f, i, j, p**).

### 4.12 Multivariate analyses

The following continuous variables were introduced: age, lymphocyte and leukocyte counts, time interval between alloHCT and vaccination, time interval between alloHCT and antibody testing, time interval between vaccination and antibody testing, and serum IgG-, IgA- and IgM levels. The following variables were introduced as categorical variables: diagnosis, sex, type of transplantation, type of vaccine, type of conditioning, immunosuppressive therapy and grade of chronic GVHD. After the first vaccination, the following factors predicted antibody response: diagnosis, time interval between alloHCT and antibody testing, time interval between alloHCT and vaccination, as well as lymphocyte count. After the second vaccination, the relevant variables were immunosuppressive therapy, severity of GVHD, time interval between alloHCT and antibody testing, interval between 1^st^ vaccination and antibody testing, serum IgG- and IgA-levels. After the third vaccination, no independent predictors remained (**Supplementary Table 6**).

None of the variables sex, age, or time of antibody testing after vaccination emerged as predictive of the induction of anti-spike-1 IgG antibodies at any time point in healthy individuals (data not shown).

## 5 Discussion

This longitudinal analysis shows that alloHCT recipients achieve robust humoral immunity after three SARS-CoV-2 vaccinations, ultimately reaching antibody levels and seropositivity rates similar to those of healthy individuals. Although some patients exhibited attenuated or delayed early responses, booster vaccination effectively compensated for these differences, emphasizing the importance of repeated antigen exposure in immunocompromised populations. Specifically, we analyzed IgG, IgM, and IgA antibodies directed against spike-1, spike-RBD and the nucleocapsid.

Our findings confirm and extend previous reports showing improved seroconversion after booster vaccination in alloHCT recipients. However, most previous studies have only examined responses up to the second vaccination (3, 7–9, 14, 15, 17, 24–27). Before vaccination, 9–15% of healthy individuals and 6-14% of alloHCT recipients already had detectable IgG antibodies, primarily against the nucleocapsid, suggesting prior SARS-CoV-2 infection. PCR results confirmed previous infection in these patients. As expected, IgM and IgA antibodies were rarely detected at baseline, as these are characteristic of the acute infection phase and do not persist in the long term (28). A key finding of this study is the delayed yet sustained antibody kinetics observed in alloHCT recipients. In contrast to IgG, anti-spike-1 and anti-spike-RBD IgM and IgA reactivities remained largely unchanged following vaccination. The slight increase in nucleocapsid-directed IgG and IgM antibodies observed in the control group likely reflects intercurrent infection rather than vaccine-induced responses. The convergence of anti-spike-1 IgG antibody prevalence between patients and controls after the third dose is clinically relevant as it suggests that immune reconstitution after alloHCT – although prolonged – can support adequate vaccine responsiveness when sufficient stimulation is provided. This may reflect gradual B-cell maturation and donor-derived immune reconstitution. Following alloHCT, immune recovery proceeds via thymic-independent peripheral expansion, followed by the re-establishment of the naïve and memory compartments. Repeated antigenic stimulation through booster vaccinations likely enhances affinity maturation and clonal expansion, thereby compensating for early deficits. Interestingly, patients were transiently more frequently anti-spike-RBD positive after the second vaccination than healthy individuals, probably indicating stronger neutralizing antibody production at those time points. We cannot explain this phenomenon. There was no increased infection rate in the patients at that time points compared to the controls, as verified by PCR and the presence of antibodies to the nucleocapsid. However, IgG anti-spike-RBD was generally lower than anti-spike-1 reactivity, which may reflect differences in assay sensitivity or cut-off thresholds. Nevertheless, this does not necessarily indicate better protection at these time points, since the protective threshold for antibody levels remains undefined (16, 18, 29, 30).

In healthy individuals, age had no significant impact on antibody response. In contrast, patients over 60 years showed significantly lower antibody levels after the second dose, which is consistent with the immunosenescence observed in transplant and non-transplant populations (7, 31, 32). However, the third vaccination substantially improved responses even in older individuals. This observation supports the use of intensified booster strategies in elderly alloHCT recipients, indicating that reduced early responsiveness does not equate to permanent non-responsiveness. Interestingly, sex did not influence antibody induction in our cohorts, despite prior reports suggesting stronger immune responses in females (33, 34). Differences in study populations, age distribution, and immunosuppressive exposure may account for this discrepancy.

Vaccine type significantly influenced serological outcomes. MRNA vaccines elicited best seroconversion after the first dose. Heterologous vaccination strategies in the follow up elicited the strongest responses, consistent with previous data (35). Both, mRNA and vector vaccines activate innate immunity; however, mRNA vaccines also induce strong B- and T-cell responses, potentially mediated through dendritic cell activation (35–38).

Interestingly, prior infection with SARS-CoV-2 before the first vaccination increased responsiveness to the first dose, but did not induce hyper-responsiveness after the second or third doses, underlining the safety of the vaccination regimen.

Underlying hematological diagnosis had a limited overall impact. While certain subgroups, such as chronic leukemia or MPN, displayed weaker intermediate responses, booster vaccination mitigated disparities. Also, there was no significant impact of donor type, although there were slower responses with mismatched transplants.

The time since transplantation had only a modest impact. Although responses were weaker after the second dose within the first 24 months post-HCT, this effect disappeared after the third. This aligns with earlier observations that early post-transplant hyporesponsiveness relates to incomplete B-cell recovery (13, 24, 39–43). In the present study, we did not observe a relationship between non-responsiveness to vaccination and the timing of vaccination after alloHCT; however, the statistical analysis may have been affected by the small number of patients. In contrast, our data indicate that early vaccination, sometimes as soon as one month after alloHCT, can induce measurable antibody responses when followed by adequate boosting. This finding supports current recommendations to vaccinate early without undue delay.

Immunosuppressive therapy impaired responses after the second vaccination, which is consistent with other reports (24, 43, 44). Notably, this negative effect was, again, largely overcome after the third dose. Clinically, this suggests that immunosuppressed patients should not be considered permanently refractory, and that they may benefit disproportionately from booster vaccination.

GVHD influenced early kinetics. Patients without GVHD mounted earlier responses, whereas those with moderate or severe GVHD exhibited delayed increases. Nevertheless, after three doses, antibody levels equalized across GVHD strata. Importantly, as also reported by others (45), we did not observe GVHD exacerbations temporally related to vaccination, which supports the safety of booster strategies in this population.

Conditioning intensity exerted a transient influence. MAC recipients demonstrated stronger responses after the second dose than RIC recipients, possibly reflecting differences in residual host immunity or immune reconstitution dynamics (24, 25, 39, 46–48). However, these differences disappeared after the third dose.

Laboratory parameters provided additional insights. Although the overall correlation between antibody reactivity and leukocyte, lymphocyte, or total IgG levels was weak, patients who remained seronegative after the third dose exhibited significantly lower lymphocyte counts and reduced IgA and IgM levels. These findings suggest that quantitative immunoglobulin deficiencies, particularly those affecting early isotype responses, may indicate impaired humoral competence.

As expected, monoclonal anti-SARS-CoV-2 therapy increased anti-spike-1 IgG but not nucleocapsid IgG reactivity. Breakthrough infections occurred despite vaccination or antibody therapy, which is consistent with reduced vaccine effectiveness against later variants such as Delta and Omicron (16, 22, 39, 45, 49). However, they were all mild.

Several limitations of our study should be acknowledged. Firstly, neutralizing antibodies were not measured directly; however, prior studies have demonstrated strong correlations between antibody titers and neutralization capacity. Protective antibody thresholds remain uncertain, especially since established cutoffs were derived from the original viral strain and may not fully apply to the Delta or Omicron variants (16, 18, 29, 30). Nevertheless, our follow-up data confirm the detection of these variants by our assay (unpublished). Secondly, T-cell immunity, another important component of antiviral defense (50, 51), was not assessed. However, the exact role of T-cell immunity in protection remains debated (52–55). Third, antibody testing intervals were not standardized and depended on outpatient visits resulting in some missing longitudinal samples. Fourthly, the healthy controls were younger and not formally matched to HCT patients; however, comparable antibody responses were observed, which is consistent with Takagi et al. (7). Finally, this was a single-center study. Nevertheless, the single-center study design ensured consistent protocol application, and reduced procedural variability. This enhanced the internal validity, and facilitated quality control across all study phases.

In conclusion, alloHCT patients can achieve humoral immunity comparable to that of healthy individuals after three doses of the SARS-CoV-2 vaccine. Early impairments related to age, vaccine type, immunosuppression, GVHD, or transplant timing can be largely overcome by booster vaccinations. While most SARS-CoV-2 infections are currently mild, they remain common, and the emergence of more virulent variants cannot be ruled out. Therefore, continued and potentially intensified vaccination strategies are particularly important for immunosuppressed individuals. However, even triple vaccination does not provide complete protection against infection.

## Data Availability

The raw data supporting the conclusions of this article will be made available by the authors, without undue reservation.

## References

[1] Ljungman P de la Camara R Mikulska M Tridello G Aguado B Zahrani MA . COVID-19 and stem cell transplantation; results from an EBMT and GETH multicenter prospective survey. Leukemia. (2021) 35:2885–94. doi: 10.1038/s41375-021-01302-5 34079042 PMC8171362

[2] Strasfeld L . COVID-19 and HSCT (Hematopoietic stem cell transplant). Best Pract Res Clin Haematol. (2022) 35:101399. doi: 10.1016/j.beha.2022.101399 36494150 PMC9547387

[3] Huang A Cicin-Sain C Pasin C Epp S Audige A Muller NJ . Antibody response to SARS-CoV-2 vaccination in patients following allogeneic hematopoietic cell transplantation. Transplant Cell Ther. (2022) 28:214:e1–e11. doi: 10.1016/j.jtct.2022.01.019 35092892 PMC8802693

[4] Asimakopoulos JV Lalou E Seferlis G Malliarou M Konstantinou E Drandakis I . Monitoring humoral response following BNT162b2 mRNA vaccination against SARS-CoV-2 in hematopoietic stem-cell transplantation patients: a single-center prospective study along with a brief review of current literature. Hematol Rep. (2024) 16:220–33. doi: 10.3390/hematolrep16020022 38651451 PMC11036264

[5] Chaekal OK Gomez-Arteaga A Chen Z Soave R Shore T Mayer S . Predictors of Covid-19 vaccination response after In-Vivo T-cell-depleted stem cell transplantation. Transplant Cell Ther. (2022) 28:618:e1–e10. doi: 10.1016/j.jtct.2022.06.012 35724850 PMC9213029

[6] Hutter-Kronke ML Neagoie A Blau IW Wais V Vuong L Gantner A . Risk factors and characteristics influencing humoral response to COVID-19 vaccination in patients after allogeneic stem cell transplantation. Front Immunol. (2023) 14:1174289. doi: 10.3389/fimmu.2023.1174289 37207199 PMC10190126

[7] Takagi E Terakura S Fujigaki H Okamoto A Miyao K Sawa M . Antibody response after third dose of COVID-19 mRNA vaccination in allogeneic hematopoietic stem cell transplant recipients is comparable to that in healthy counterparts. Int J Hematol. (2023) 118:462–71. doi: 10.1007/s12185-023-03648-1 37561340

[8] Wu X Wang L Shen L He L Tang K . Immune response to vaccination against SARS-CoV-2 in hematopoietic stem cell transplantation and CAR T-cell therapy recipients. J Hematol Oncol. (2022) 15:81. doi: 10.1186/s13045-022-01300-9 35710431 PMC9200932

[9] Bordat J Maury S Leclerc M . Allogeneic hematopoietic stem cell transplantation in the COVID-19 era. Front Immunol. (2023) 14:1100468. doi: 10.3389/fimmu.2023.1100468 36911678 PMC9993088

[10] Yuan M Wilson IA . Structural immunology of SARS-CoV-2. Immunol Rev. (2025) 329:e13431. doi: 10.1111/imr.13431 39731211 PMC11727448

[11] Premkumar L Segovia-Chumbez B Jadi R Martinez DR Raut R Markmann A . The receptor binding domain of the viral spike protein is an immunodominant and highly specific target of antibodies in SARS-CoV-2 patients. Sci Immunol. (2020) 5:1–9. doi: 10.1126/sciimmunol.abc8413 32527802 PMC7292505

[12] Ali H Ngo D Aribi A Arslan S Dadwal S Marcucci G . Safety and tolerability of SARS-CoV2 emergency-use authorized vaccines for allogeneic hematopoietic stem cell transplant recipients. Transplant Cell Ther. (2021) 27:938:e1–e6. doi: 10.1016/j.jtct.2021.07.008 34274492 PMC8280601

[13] Nikoloudis A Neumann IJ Buxhofer-Ausch V Machherndl-Spandl S Binder M Kaynak E . Successful SARS-CoV-2 mRNA vaccination program in allogeneic hematopoietic stem cell transplant recipients-a retrospective single-center analysis. Vaccines (Basel). (2023) 11:1–12. doi: 10.3390/vaccines11101534 37896938 PMC10611175

[14] Chevallier P Jullien M Peterlin P Garnier A Le Bourgeois A Coste-Burel M . Effectiveness of a third dose of BNT162b2 anti-SARS-CoV-2 mRNA vaccine over a 6-month follow-up period in allogenic hematopoietic stem cells recipients. Hematol Oncol. (2022) 40:1097–9. doi: 10.1002/hon.3006 35468662 PMC9087413

[15] Leclerc M Redjoul R Le Bouter A Beckerich F Robin C Parinet V . Determinants of SARS-CoV-2 waning immunity in allogeneic hematopoietic stem cell transplant recipients. J Hematol Oncol. (2022) 15:27. doi: 10.1186/s13045-022-01250-2 35303906 PMC8931584

[16] Canti L Arien KK Desombere I Humblet-Baron S Pannus P Heyndrickx L . Antibody response against SARS-CoV-2 Delta and Omicron variants after third-dose BNT162b2 vaccination in allo-HCT recipients. Cancer Cell. (2022) 40:335–7. doi: 10.1016/j.ccell.2022.02.005 35172125 PMC8847067

[17] Einarsdottir S Martner A Nicklasson M Wiktorin HG Arabpour M Tornell A . Reduced immunogenicity of a third COVID-19 vaccination among recipients of allogeneic hematopoietic stem cell transplantation. Haematologica. (2022) 107:1479–82. doi: 10.3324/haematol.2021.280494 35236057 PMC9152965

[18] Tamari R Politikos I Knorr DA Vardhana SA Young JC Marcello LT . Predictors of humoral response to SARS-CoV-2 vaccination after hematopoietic cell transplantation and CAR T-cell therapy. Blood Cancer Discov. (2021) 2:577–85. doi: 10.1158/2643-3230.bcd-21-0142 34778798 PMC8580614

[19] Jagasia MH Greinix HT Arora M Williams KM Wolff D Cowen EW . National institutes of health consensus development project on criteria for clinical trials in chronic graft-versus-host disease: I. The 2014 diagnosis and staging working group report. Biol Blood Marrow Transplant. (2015) 21:389–401:e1. doi: 10.1016/j.bbmt.2014.12.001 25529383 PMC4329079

[20] Klein R Marx A Strobel P Schalke B Nix W Willcox N . Autoimmune associations and autoantibody screening show focused recognition in patient subgroups with generalized myasthenia gravis. Hum Immunol. (2013) 74:1184–93. doi: 10.1016/j.humimm.2013.06.020 23792059

[21] Nachtigall C Wirtz M . Wahrscheinlichkeitsrechnung Und Inferenzstatistik. Weinheim: Juventa Verlag (2004).

[22] Tsushima T Terao T Narita K Fukumoto A Ikeda D Kamura Y . Antibody response to COVID-19 vaccine in 130 recipients of hematopoietic stem cell transplantation. Int J Hematol. (2022) 115:611–5. doi: 10.1007/s12185-022-03325-9 35426579 PMC9011370

[23] Savas EM Yildiz S Ozkurt ZN Baltaci Z Guzel Tunccan O Yegin ZA . COVID-19 vaccine response in Allo-HSCT recipients: insights from a real-world prospective cohort study. Vaccines (Basel). (2025) 13:1–11. doi: 10.3390/vaccines13070726 PMC12299849 40733703

[24] Meejun T Srisurapanont K Manothummetha K Thongkam A Mejun N Chuleerarux N . Attenuated immunogenicity of SARS-CoV-2 vaccines and risk factors in stem cell transplant recipients: a meta-analysis. Blood Adv. (2023) 7:5624–36. doi: 10.1182/bloodadvances.2023010349 37389818 PMC10514108

[25] Le Bourgeois A Coste-Burel M Guillaume T Peterlin P Garnier A Imbert BM . Interest of a third dose of BNT162b2 anti-SARS-CoV-2 messenger RNA vaccine after allotransplant. Br J Haematol. (2022) 196:e38–e40. doi: 10.1111/bjh.17911 34671982 PMC8653164

[26] Redjoul R Le Bouter A Parinet V Fourati S Maury S . Antibody response after third BNT162b2 dose in recipients of allogeneic HSCT. Lancet Haematol. (2021) 8:e681–e3. doi: 10.1016/s2352-3026(21)00274-x 34487683 PMC8415894

[27] Attolico I Tarantini F Carluccio P Musto P . Serological response following anti-SARS-CoV-2 vaccination in hematopoietic stem cell transplantation patients depends upon time from transplant, type of transplant and "booster" dose. Haematologica. (2022) 107:1218. doi: 10.3324/haematol.2022.280619 35045696 PMC9052926

[28] Amellal H Assaid N Charoute H Akarid K Maaroufi A Ezzikouri S . Kinetics of specific anti-SARS-CoV-2 IgM, IgA, and IgG responses during the first 12 months after SARS-CoV-2 infection: a prospective longitudinal study. PloS One. (2023) 18:e0288557. doi: 10.1371/journal.pone.0288557 37437051 PMC10337929

[29] Khoury DS Cromer D Reynaldi A Schlub TE Wheatley AK Juno JA . Neutralizing antibody levels are highly predictive of immune protection from symptomatic SARS-CoV-2 infection. Nat Med. (2021) 27:1205–11. doi: 10.1038/s41591-021-01377-8 34002089

[30] Krammer F . A correlate of protection for SARS-CoV-2 vaccines is urgently needed. Nat Med. (2021) 27:1147–8. doi: 10.1038/s41591-021-01432-4 34239135

[31] Collier DA Ferreira I Kotagiri P Datir RP Lim EY Touizer E . Age-related immune response heterogeneity to SARS-CoV-2 vaccine BNT162b2. Nature. (2021) 596:417–22. doi: 10.1038/s41586-021-03739-1 34192737 PMC8373615

[32] Bonnet B Chabrolles H Archimbaud C Brebion A Cosme J Dutheil F . Decline of humoral and cellular immune responses against SARS-CoV-2–6 months after full BNT162b2 vaccination in hospital healthcare workers. Front Immunol. (2022) 13:842912. doi: 10.3389/fimmu.2022.842912 35309363 PMC8926062

[33] Fink AL Klein SL . Sex and gender impact immune responses to vaccines among the elderly. Physiol (Bethesda). (2015) 30:408–16. doi: 10.1152/physiol.00035.2015 26525340 PMC4630198

[34] Takahashi T Ellingson MK Wong P Israelow B Lucas C Klein J . Sex differences in immune responses that underlie COVID-19 disease outcomes. Nature. (2020) 588:315–20. doi: 10.1038/s41586-020-2700-3 32846427 PMC7725931

[35] Meyer T Ihorst G Bartsch I Zeiser R Wasch R Bertz H . Cellular and humoral SARS-CoV-2 vaccination responses in 192 adult recipients of allogeneic hematopoietic cell transplantation. Vaccines (Basel). (2022) 10:1–11. doi: 10.3390/vaccines10111782 36366291 PMC9699205

[36] Teijaro JR Farber DL . COVID-19 vaccines: modes of immune activation and future challenges. Nat Rev Immunol. (2021) 21:195–7. doi: 10.1038/s41577-021-00526-x 33674759 PMC7934118

[37] Zhu J Huang X Yang Y . Innate immune response to adenoviral vectors is mediated by both Toll-like receptor-dependent and -independent pathways. J Virol. (2007) 81:3170–80. doi: 10.1128/jvi.02192-06 17229689 PMC1866082

[38] Li C Lee A Grigoryan L Arunachalam PS Scott MKD Trisal M . Mechanisms of innate and adaptive immunity to the Pfizer-BioNTech BNT162b2 vaccine. Nat Immunol. (2022) 23:543–55. doi: 10.1038/s41590-022-01163-9 35288714 PMC8989677

[39] Maillard A Redjoul R Klemencie M Labussiere Wallet H Le Bourgeois A D'Aveni M . Antibody response after 2 and 3 doses of SARS-CoV-2 mRNA vaccine in allogeneic hematopoietic cell transplant recipients. Blood. (2022) 139:134–7. doi: 10.1182/blood.2021014232 34818411 PMC8616709

[40] Morsink LM van Doesum J Choi G Hazenberg CLE Biswana A Meppelink F . Robust COVID-19 vaccination response after allogeneic stem cell transplantation using post transplantation cyclophosphamide conditioning. Blood Cancer J. (2022) 12:6. doi: 10.1038/s41408-021-00605-1 35022420 PMC8754065

[41] Mamez AC Pradier A Giannotti F Petitpas A Urdiola MF Vu DL . Antibody responses to SARS-CoV2 vaccination in allogeneic hematopoietic stem cell transplant recipients. Bone Marrow Transplant. (2021) 56:3094–6. doi: 10.1038/s41409-021-01466-9 34584239 PMC8477622

[42] Mackall C Fry T Gress R Peggs K Storek J Toubert A . Background to hematopoietic cell transplantation, including post transplant immune recovery. Bone Marrow Transplant. (2009) 44:457–62. doi: 10.1038/bmt.2009.255 19861978

[43] Ogonek J Kralj Juric M Ghimire S Varanasi PR Holler E Greinix H . Immune reconstitution after allogeneic hematopoietic stem cell transplantation. Front Immunol. (2016) 7:507. doi: 10.3389/fimmu.2016.00507 27909435 PMC5112259

[44] Majcherek M Matkowska-Kocjan A Szymczak D Karasek M Szeremet A Kiraga A . Two doses of BNT162b2 mRNA vaccine in patients after hematopoietic stem cell transplantation: humoral response and serological conversion predictors. Cancers (Basel). (2022) 14:1–14. doi: 10.3390/cancers14020325 35053487 PMC8773492

[45] Pinana JL Martino R Vazquez L Lopez-Corral L Perez A Chorao P . SARS-CoV-2-reactive antibody waning, booster effect and breakthrough SARS-CoV-2 infection in hematopoietic stem cell transplant and cell therapy recipients at one year after vaccination. Bone Marrow Transplant. (2023) 58:567–80. doi: 10.1038/s41409-023-01946-0 PMC9974060 36854892

[46] Mori Y Uchida N Harada T Katayama Y Wake A Iwasaki H . Predictors of impaired antibody response after SARS-CoV-2 mRNA vaccination in hematopoietic cell transplant recipients: a Japanese multicenter observational study. Am J Hematol. (2023) 98:102–11. doi: 10.1002/ajh.26769 36260658 PMC9874814

[47] Shem-Tov N Yerushalmi R Danylesko I Litachevsky V Levy I Olmer L . Immunogenicity and safety of the BNT162b2 mRNA COVID-19 vaccine in haematopoietic stem cell transplantation recipients. Br J Haematol. (2022) 196:884–91. doi: 10.1111/bjh.17918 34713441 PMC8652777

[48] Yeshurun M Pasvolsky O Shargian L Yahav D Ben-Zvi H Rubinstein M . Humoral serological response to the BNT162b2 vaccine after allogeneic haematopoietic cell transplantation. Clin Microbiol Infect. (2022) 28:303:e1–e4. doi: 10.1016/j.cmi.2021.10.007 34715348 PMC8553414

[49] Barda N Dagan N Cohen C Hernan MA Lipsitch M Kohane IS . Effectiveness of a third dose of the BNT162b2 mRNA COVID-19 vaccine for preventing severe outcomes in Israel: an observational study. Lancet. (2021) 398:2093–100. doi: 10.1016/s0140-6736(21)02249-2 34756184 PMC8555967

[50] Liebers N Speer C Benning L Bruch PM Kraemer I Meissner J . Humoral and cellular responses after COVID-19 vaccination in anti-CD20-treated lymphoma patients. Blood. (2022) 139:142–7. doi: 10.1182/blood.2021013445 34669919 PMC8530768

[51] Sette A Crotty S . Adaptive immunity to SARS-Cov-2 and COVID-19. Cell. (2021) 184:861–80. doi: 10.1016/j.cell.2021.01.007 33497610 PMC7803150

[52] Lindemann M Klisanin V Thummler L Fisenkci N Tsachakis-Muck N Ditschkowski M . Humoral and cellular vaccination responses against SARS-CoV-2 in hematopoietic stem cell transplant recipients. Vaccines (Basel). (2021) 9:1–15. doi: 10.3390/vaccines9101075 34696183 PMC8537291

[53] Ram R Hagin D Kikozashvilli N Freund T Amit O Bar-On Y . Safety and immunogenicity of the BNT162b2 mRNA COVID-19 vaccine in patients after allogeneic HCT or CD19-based CART therapy-a single-center prospective cohort study. Transplant Cell Ther. (2021) 27:788–94. doi: 10.1016/j.jtct.2021.06.024 34214738 PMC8242200

[54] Harrington P Doores KJ Saha C Saunders J Child F Dillon R . Repeated vaccination against SARS-CoV-2 elicits robust polyfunctional T cell response in allogeneic stem cell transplantation recipients. Cancer Cell. (2021) 39:1654. doi: 10.1016/j.ccell.2021.10.002 34906318 PMC8667332

[55] Einarsdottir S Martner A Waldenstrom J Nicklasson M Ringlander J Arabpour M . Deficiency of SARS-CoV-2 T-cell responses after vaccination in long-term allo-HSCT survivors translates into abated humoral immunity. Blood Adv. (2022) 6:2723–30. doi: 10.1182/bloodadvances.2021006937 35286374 PMC8923719

