# Supplementary material for: Humoral responses to SARS-CoV-2 vaccination in allogeneic hematopoietic cell transplantation patients resemble that of healthy individuals
Source: Front Immunol. 2026 Jul 1;17:1829281. doi: 10.3389/fimmu.2026.1829281 (PMC13368556; doi:10.3389/fimmu.2026.1829281)
Supplement: Supplementary file 1 [file DataSheet1.docx]

**Supplementary data**

**Table S1** Demographic, clinical and laboratory parameters in allogeneic HCT recipients with different hematological disorders analyzed for antibo­dies to SARS-CoV-2

A)

| Diseases^*)^ | patients analyzed (n) | sex m/f (n) | Conditioning therapy RIC/MAC (n) | IS at time of serologi­cal analy­sis n (%) | HCT | | | cGVHD | | | |
| --- | --- | --- | --- | --- | --- | --- | --- | --- | --- | --- | --- |
|  |  |  |  |  | unrelated | related | none^**)^ | none | mild | moderate | severe |

|  | |  |  |  |  | id | MM | id | haplo |  |  |  |  |  |
| --- | --- | --- | --- | --- | --- | --- | --- | --- | --- | --- | --- | --- | --- | --- |
| **total** | | 354 | 195/159 | 177/177 | 54 (15) | 177 | 50 | 93 | 25 | 9 | 242 | 72 | 28 | 12 |
| 1 | acute leukemia | 196 | 98/98 | 86/110 | 28 (14) | 93 | 33 | 50 | 17 | 3 | 136 | 41 | 14 | 5 |
| 2 | chronic leukemia | 37 | 21/16 | 13/24 | 1 (3) | 17 | 1 | 15 | 4 | 0 | 28 | 8 | 1 | 0 |
| 3 | MDS | 46 | 22/24 | 32/14 | 11 (24) | 25 | 7 | 11 | 2 | 1 | 25 | 7 | 8 | 6 |
| 4 | MPN | 27 | 18/9 | 20/7 | 9 (33) | 13 | 7 | 5 | 0 | 2 | 17 | 8 | 2 | 0 |
| 5 | B NHL | 11 | 9/2 | 8/3 | 1 (9) | 8 | 0 | 3 | 0 | 0 | 4 | 5 | 1 | 1 |
| 6 | CMML | 7 | 3/4 | 7/0 | 1 (14) | 5 | 1 | 0 | 0 | 1 | 6 | 1 | 0 | 0 |
| 7 | plasma cell disorders | 7 | 7/0 | 5/2 | 0 | 4 | 0 | 3 | 0 | 0 | 7 | 0 | 0 | 0 |
| 8 | T NHL | 8 | 5/3 | 4/4 | 2 (25) | 3 | 1 | 3 | 0 | 1 | 7 | 1 | 0 | 0 |
| 9 | other malignant hematological disorders | 7 | 6/1 | 1/6 | 1 (14) | 4 | 0 | 1 | 1 | 1 | 4 | 1 | 2 | 0 |
| 10 | non-malignant hematological and other disorders | 8 | 6/2 | 1/7 | 0 | 5 | 0 | 2 | 1 | 0 | 8 | 0 | 0 | 0 |

B)

| Diseases^*)^ | | age (years) | Serum samp­les per patient (n) | 1^st^ vacc in re­lation to alloHCT (months) | 1^st^ serum sample in re­lation to alloHCT (months) | Time be­tween 1^st^ vacc and antibody testing (days) | laboratory values at time of first analysis | | | | |
| --- | --- | --- | --- | --- | --- | --- | --- | --- | --- | --- | --- |
|  |  |  |  |  |  |  | Lympho-cytes (G/l) | Leukocytes (G/l) | Quantitative serum immunglobulins (mg%) | | |
|  |  |  |  |  |  |  |  |  | IgG | IgA | IgM |
|  |  | mean+SD | | | | | | | | | |
|  |  | median | | | | | | | | | |
|  |  | range | | | | | | | | | |
| **total** | | 57+14 | 3+2 | 87.3+83.4 | 94.0+88.5 | 22.6+20.9 | 2.68+14.52 | 7.174+15.439 | 916.9+408.3 | 143.7+99.0 | 90.5+75.3 |
|  |  | 60 | 2 | 66.3 | 68.0 | 17 | 2.00 | 6.010 | 897.0 | 128.0 | 73.0 |
|  |  | 18-93 | 1-14 | -7.7-354.3 | -8.0-361.0 | 1-138.6 | 0-260.88 | 20-277.970 | 37.0-3.413 | 2-467 | 9-643 |
| 1 | acute leukemia | 56+14 | 3+2 | 90.0+72.1 | 81.0+73.8 | 95+73.7 | 1.95+1.32 | 6.611.2+3.185 | 915.4+414.7 | 146.2+101.8 | 89.3+82.4 |
|  |  | 58 | 2 | 71.5 | 66.9 | 73 | 1.68 | 6.210 | 899.0 | 130.0 | 71.5 |
|  |  | 18-93 | 1-14 | 3.6-290.3 | -7.7-324.8 | 1-265 | 0.00-13.9 | 20-32.740 | 129.0-3413.0 | 2.0-440.0 | 9.0-643.0 |
| 2 | chronic leukemia | 60+9 | 2+1 | 218.9+102.2 | 200.8+115.1 | 128+60.9 | 0.86+1.72 | 6.216+2.135 | 904.0+247.1 | 166.6+95.2 | 81.4+43.3 |
|  |  | 60 | 2 | 259.6 | 248.0 | 147 | 1.88 | 5.800 | 936.0 | 142.5 | 74.5 |
|  |  | 34-82 | 1-8 | 5.0-354.3 | 5.1-12.0 | 4-201 | 0.38-4.92 | 3.430-12.160 | 261.0-1.375.0 | 2.0-398.0 | 9.0-210.0 |
| 3 | MDS | 63+10 | 3+2 | 104.6+67.5 | 78.0+70.7 | 99+71.5 | 2.00+1.31 | 5.707+2.407 | 1.064.7+547.0 | 132.3+72.2 | 103.3+68.8 |
|  |  | 64 | 2 | 107.6 | 54.3 | 104 | 1.84 | 5.445 | 1.071.0 | 139.5 | 83.0 |
|  |  | 35-80 | 1-12 | 6.5-282.9 | 0.9-283.7 | 2-228 | 0.01-6.54 | 110-11.570 | 37.0-3.284.0 | 6.0-338.0 | 38.0-382.0 |
| 4 | MPN | 64+5 | 4+3 | 53.9+56.0 | 60.5+77.3 | 66+60.7 | 1.02+0.60 | 5.182+1.759 | 732.0+192.6 | 103.7+66.1 | 109.4+95.9 |
|  |  | 65 | 4 | 19.0 | 15.6 | 44 | 0.81 | 4.945 | 709.5 | 92.5 | 69.0 |
|  |  | 54-73 | 1-12 | 3.0-166.4 | 0.9-276.0 | 9-216 | 0.23-2.30 | 1.810-8.720 | 345.0-1118.0 | 2.0-290.0 | 10.0-437.0 |
| 5 | B NHL | 58+13 | 4+3 | 90.8+54.1 | 85.2+69.1 | 72+57.8 | 2.65+1.96 | 7.743+3.369 | 859.0+152.4 | 192.7+124.2 | 78.3+28.8 |
|  |  | 58 | 4 | 90.9 | 61.3 | 52 | 2.19 | 6.970 | 772.0 | 164.0 | 75.0 |
|  |  | 26-79 | 1-10 | 16.9-177.1 | 12.5-223.1 | 12-163 | 1.16-8.33 | 4.400-17.290 | 676.0-1.080.0 | 74.0-467.0 | 42.0-114.0 |
| 6 | CMML | 63+7 | 4+3 | 84.9+49.7 | 73.9+53.5 | 73+57.2 | 1.65+0.67 | 6.073+1.784 | 1.178.8+165.3 | 187.3+68.7 | 120.8+10.2 |
|  |  | 62 | 2 | 81.4 | 76.0 | 81 | 1.54 | 5.730 | 1.102.0 | 170.5 | 117.0 |
|  |  | 55-71 | 1-6 | 13.2-164.4 | 9.7-164.4 | 1-153 | 0.75-2.64 | 3.960-9.730 | 1.053-1.458 | 116.0-292.0 | 112.0-137.0 |
| 7 | plasma cell disorders | 61+11 | 3+1 | 82.8+58.7 | 80.7+57.0 | 115+60.7 | 1.59+0.49 | 5.390+960 | 835.3+452.6 | 134.5+111.1 | 88.7+69.5 |
|  |  | 65 | 3 | 70.9 | 65.8 | 123 | 1.42 | 5.450 | 901.0 | 74.0 | 76.5 |
|  |  | 44-68 | 1-4 | 16.3-202.0 | 16.5-206.6 | 7-195 | 1.06-2.48 | 3.770-6.560 | 229.0-1.356.0 | 38.0-335.0 | 11.0-225.0 |
| 8 | T NHL | 54+17 | 2+3 | 145.1+5.2 | 104.2+54.8 | 111+36.5 | 2.41+1.25 | 7.544+1.754 | 950.3+259.0 | 92.5+53.2 | 92.3+31.6 |
|  |  | 59 | 1 | 145.1 | 125.0 | 111 | 1.25 | 7.940 | 1.045.0 | 89.5 | 85.5 |
|  |  | 20-71 | 1-7 | 139.9-150.2 | 5.4-154.5 | 75-148 | 1.06-4.26 | 5.100-9.740 | 568.0-1.216.0 | 9.0-189.0 | 47.0-138.0 |
| 9 | other malig-nant hematological disor­ders | 58+17 | 4+3 | 93.4+120.4 | 112.0+121.0 | 71+61.1 | 1.21+0.54 | 5.456+2.219 | 844.9+349.1 | 110.1+57.0 | 72.0+58.5 |
|  |  | 61 | 3 | 34.0 | 40.0 | 49 | 1.21 | 4.390 | 844.0 | 107.0 | 47.0 |
|  |  | 26-75 | 1-8 | 5.6-300.7 | 6.0-306.5 | 13-174 | 0.26-2.09 | 2.570-8.840 | 271.0-1.380 | 12.0-188.0 | 9.0-200.1 |
| 10 | non-malig-nant hematological and other disor­ders | 32+13 | 3+3 | 134.8+109.0 | 103.6+98.0 | 88+63.9 | 3.31+4.48 | 8.027+7.270 | 749.0+186.1 | 131.3+81.5 | 47.0+11.6 |
|  |  | 29 | 2 | 87.1 | 64.3 | 57 | 1.89 | 5.350 | 663.0 | 146.0 | 55.0 |
|  |  | 20-62 | 1-8 | 7.1-291.8 | 9.0-293.7 | 42-199 | 0.78-15.11 | 4.750-27.230 | 546.0-1.138.0 | 25.0-251 | 28.0-58.0 |

MDS-myelodysplastic syndrome; MPN-myeloproliferative neoplasms; NHL-Non-Hodgkin lymphoma; CMML-chronic myelomonocytic leukemia

RIC-reduced intensity conditioning; MAC-myeloablative conditioning; cGVHD chronic graft-versus-host disease

n-number

IS-immunosuppressive therapy (cyclosporine, methotrexate, tacrolimus or mycophenolate mofetil)

id-identical; haplo id-haploidentical; MM-mismatch

^*)^ for detailed subgrouping see Supplementary data Table S2

^**)^ at time of first analysis

**Table S2 Underlying diagnosis in alloHCT recipients analyzed for anti-SARS-CoV-2 reactivity after vaccination**

|  | | | patients analyzed | Sex m/f |
| --- | --- | --- | --- | --- |
|  |  |  | number | |
| 1) | | acute leukemia (total) | 196 | 98/98 |
|  |  | T- and B-ALL | 45 | 28/17 |
|  |  | AML/sec.AML | 151 | 70/81 |
| 2) | | chronic leukemia (total) | 37 | 21/16 |
|  |  | CLL | 2 | 1/1 |
|  |  | CML | 35 | 20/15 |
| 3) | | MDS (total) | 46 | 22/24 |
| 4) | | MPN (total) | 27 | 18/9 |
| 5) | | B-NHL (total) | 11 | 9/2 |
| 6) | | CMML (total) | 7 | 3/4 |
| 7) | | plasma cell disorders (total) | 7 | 7/0 |
|  |  | Multiple myeloma, plasmocytoma | 6 | 6/0 |
|  |  | Plasma cell leukemia | 1 | 1/0 |
| 8) | | T-NHL (total) | 8 | 5/3 |
| 9) | other malignant hematological dis­orders (total) | | 7 | 6/1 |
| BPDCN | | | 3 | 3/0 |
| Hodgkin lymphoma | | | 2 | 2/0 |
| T-PLL | | | 2 | 1/1 |
| 10) | | non-malignant hematological and  other disorders (total) | 8 | 6/2 |
|  |  | Aplastic anemia/SAA | 3 | 2/1 |
|  |  | Thalassemia major | 1 | 1/0 |
|  |  | PNH | 1 | 1/0 |
|  |  | Diamond Blackfan anemia | 1 | 0/1 |
|  |  | Metachromatic leukodystrophy | 1 | 1/0 |
|  |  | Ewing sarcoma | 1 | 1/0 |
| **total** | | | 354 | 195/159 |

ALL-acute lymphocytic leukemia; AML-acute myeloid leukemia; CLL-chronic lymphocytic leukemia; CML-chronic myeloid leukemia; CMML-chronic myelomonocytic leukemia; MDS-myelodysplastic syndrome; MPN-myeloprolife­rative neoplasms; NHL-Non-Hodgkin-lymphoma; BPDCN-blastic plasmacytoid dendritic cell neoplasm; PLL-prolymphocytic leukemia; SAA-severe aplastic anemia; PNH-paroxysmal nocturnal hemoglobinuria.

**Table S3** Prevalence of SARS-CoV-2 infection verified by PCR before and after vaccinations in patients after alloHCT and healthy controls.

| Time point | patients | Healthy individuals |
| --- | --- | --- |
|  | Number positive/number tested (%) | |
| Before 1. vaccination | 24/146 (16) | 35/449 (8) ^**)^ |
| between 1. and 2. vaccina­tion | 15/104 (14) | 2/71 (3) ^*)^ |
| between 2. and 3. vaccina­tion | 7/262 (3)^a)^ | 4/193 (1)^c)^ |
| after 3. vaccination | 4/123 (3)^b)^ | 13/134 (10) ^***)d)^ |

Significantly lower prevalence compared to patients with hematological disorders: *) p < 0.05; **) p < 0.01

Significantly higher prevalence compared to patients with hematological disorders: ***) p < 0.001

Significantly lower prevalence compared to the time point before 1^st^ vaccination: ^a)^ p < 0.00001; ^b)^ p < 0.0001 ^c)^ p < 0.01

Significantly higher prevalence compared to the time point after 2^nd^ vaccination: ^d)^ p < 0.01

**Table S4** Prevalence of IgG-anti-spike-1 antibodies in alloHCT recipients after vaccination in relation to the type of vaccine

| 1^st^ vaccination |  |  | Number patients | Number (%) IgG-anti-spike-1 antibody positive |
| --- | --- | --- | --- | --- |
| B |  |  | 76 | 23 (30) |
| A |  |  | 15 | 0 |
| M |  |  | 5 | 1 (20) |
| J |  |  | 3 | 1 (33 |
| 1^st^ vaccination | 2^nd^ vaccination |  |  |  |
| B | B |  | 182 | 110 (60) |
| B | A |  | 2 | 2 (100) |
| B | M |  | 1 | 1 (100) |
| A | A |  | 19 | 11 (58) |
| A | B |  | 20 | 16 (80) |
| A | M |  | 1 | 1 (100) |
| M | M |  | 13 | 10 (77) |
| 1^st^ vaccination | 2^nd^ vaccination | 3^rd^ vaccination |  |  |
| B | B | B | 64 | 59 (92) |
| B | B | M or A | 16 | 13 (81) |
| A | A | B or M | 8 | 8 (100) |
| A | B | B | 6 | 6 (100) |
| M | M | M | 3 | 3 (100) |
| M | M | B | 5 | 5 (100) |

B = mRNA-vaccine BNT162b2 (Biontech); M = mRNA-1273 (Moderna); A = vector-vaccine ChAdOx1-S (Astra­Zeneca); J = vector-vaccine Ad26.COV2-S (Johnson&Johnson)

Discrepancies in the numbers in table S3 are explained by the fact that the type of vaccine could not be evaluated for all patients at each time point.

**Table S5** Prevalence of IgG-anti-spike-1-antibodies in patients with different hematological disorders and healthy individuals before and after vaccination with SARS-CoV-2

| Diagnosis | Number of vaccinations | | | |
| --- | --- | --- | --- | --- |
|  | Before^a)^ | 1^st^ | 2^nd^ | 3^rd^ |
|  | Number anti-spike-1 positive / number tested (%) | | | |
| Acute leukemia | 5 / 72 (7) | 16 / 56 (29) ^**)^ | 89 / 138 (64) ^****) b)^ | 67 / 70 (96) ^****) b)^ |
| Chronic leukemia | 1 / 10 (10) | 0 / 5 (0) | 24 / 29 (83) ^***)^ | 9 / 10 (90) ^**)^ |
| MDS | 1 / 17 (6) ^b)^ | 2 / 11 (18) ^b)^ | 22 / 33 (67) ^****)^ | 10 / 11 (91) ^****)^ |
| MPN | 0 / 9 (0) | 6 / 17 (35) | 8 / 24 (33) | 15 / 16 (94) ^****)^ |
| B-NHL | 0 / 3 (0) | 3 / 7 (43) ^c)^ | 6 / 9 (67) ^c)^ | 3 / 4 (75) ^c)^ |
| CMML | 0 / 2 (0) | 1 / 2 (50) ^c)^ | 3 / 5 (60) ^c)^ | 2 / 2 (100) ^c)^ |
| Plasma cell disor­ders | 0 | 0 / 1 (0) ^c)^ | 5 / 7 (71) ^c)^ | 3 / 4 (75) ^c)^ |
| T-NHL | 0 / 3 (0) | 1 / 2 (50) ^c)^ | 5 / 6 (83) ^*)^ | 1 / 1 (100) ^c)^ |
| Other malignant hematological dis­orders | 0 / 2 (0) | 0 / 2 (0) ^c)^ | 3 / 5 (60) ^c)^ | 2 / 3 (67) ^c)^ |
| Non-malignant hematological and other disorders | 0 / 3 (0) | 0 / 1 (0) ^c)^ | 4 / 6 (67) ^c)^ | 2 / 2 (100) ^c)^ |
| Healthy individuals | 23 / 435 (5) | 10 / 36 (28) ^***)^ | 79 / 135 (59) ^****)^ | 109 / 111 (98) ^****)^ |

^a)^ Excluding patients with COVID-19 infection verified by PCR

^b)^ One antibody positive patient was treated with anti-SARS-CoV2 antibodies at the time of antibody testing

^c)^ Number of patients was too low to calculate significance levels

Significant difference compared to before 1^st^ vaccination: * p < 0.05; ** p < 0.01; *** p < 0.001; **** p < 0.0001.

There were no significant differences between patients with different hematological disorders and healthy controls at any time point.

**Table S6** Multivariate analysis of different parameters with respect to their prediction of serological response to SARS-CoV-2 vaccination in relation to the number of vaccinations

|  | 1^st^ vaccination | | 2^nd^ vaccination | | 3^rd^ vaccination | |
| --- | --- | --- | --- | --- | --- | --- |
|  | F-value | p | F-value | p | F-value | p |
| diagnosis | 2.278 | **0.023** | 1.248 | 0.266 | 0.388 | 0.936 |
| sex | 1.145 | 0.389 | 0.468 | 0.495 | 1.539 | 0.413 |
| type of transplantation | 0.944 | 0.601 | 0.063 | 0.802 | 1.343 | 0.47 |
| type of conditioning | 1.195 | 0.346 | 1.169 | 0.281 | 0.724 | 0.741 |
| immunosuppressive therapy | 0.926 | 0.622 | 7.607 | **0.006** | 0.56 | 0.838 |
| grade of cGVHD | 1.109 | 0.423 | 7.743 | **0.006** | 0.942 | 0.627 |
| type of vaccine | 1.662 | 0.106 | 1.717 | 0.192 | 0.581 | 0.826 |
| time interval between alloHCT and antibody testing | 2.005 | **0.044** | 19.603 | **0.000** | 1.321 | 0.477 |
| time interval between allo HCT and vaccination | 1.975 | **0.048** | 18.765 | **0.000** | 1.369 | 0.461 |
| time interval between vaccination and antibody testing | 1.452 | 0.182 | 18.686 | **0.000** | 1.25 | 0.5 |
| lymphocyte count | 3.592 | **0.001** | 0.888 | 0.347 | 0.408 | 0.926 |
| leukocyte count | 0.828 | 0.739 | 0.03 | 0.862 | 2.352 | 0.264 |
| IgG | 0.848 | 0.597 | 6.735 | **0.010** | 1.028 | 0.587 |
| IgA | 1.793 | 0.076 | 13.624 | **0.000** | 2.579 | 0.238 |
| IgM | 0.950 | 0.595 | 2.692 | 0.103 | 0.498 | 0.876 |

F-values and significances (p) are given; p < 0.05 is considered as statistically significant.

**Figure S1**

**Figure S1 Comparison of IgG-spike-1 antibody reactivity in alloHCT patients without and with COVID-19 infection prior to the first vaccination at different time points.**

AU: arbitrary units; ns: not significant

Median + 95% CI is given.

**Figure S2**

**Figure S2** **Correlation between time after vaccinations (in days) and IgG-anti-spike-1 antibody production in healthy individuals (A) and patients with hematological disorders after alloHCT (B) in relation to the number of vaccinations.**

Individual values are given. R = correlation coefficient (Spearman’s rank); p = significance level. AU: arbitrary units

---- = normal range

**Figure S3**

**Figure S3** **Relation between anti-spike-1 IgG antibody reactivity after vaccination and type of alloHCT in patients with hematological disorders.**

Id - identical; MM - mismatched; haplo - haploidentical

Significance levels as compared to 1^st^ vaccination: ** p < 0.01; **** p < 0.0001

AU: arbitrary units; ns: not significant

Median + 95% CI is given.

**Figure S4**

**Figure S4** **Relation between intensity of conditioning and anti-spike-1 IgG antibody reactivity (A), serum IgG levels (B), lymphocyte count (C) and leukocytes (D) after vaccination in pa­tients with hematological disorders after alloHCT.**

🞏 = reduced-intensity conditioning; ◼ = myeloablative conditioning

AU: arbitrary units; ns: not significant

Median + 95% CI is given.

**Figure S5**

**Figure S5 Influence of ongoing immunosuppressive therapy (A) and severity of GVHD (B) on the induc­tion of anti-spike-1 IgG antibodies after vaccination in patients after alloHCT**

IS: immunosuppressive therapy; 0: without IS

24 patients were under immunosuppressive therapy after the 2^nd^ vaccination (tacrolimus n=14, mycophenolate mofetil n=6, others n=4).There were no significant differences in anti-spike-1 reactivity between patients with the different therapeutic regimes.

1: no GVHD; 2: mild GVHD; 3: moderate GVHD; 4: severe GVHD (after 3^rd^ vaccination only one patient had severe GVHD, i.e. statistical analysis could not be performed).

AU: arbitrary units; ns: not significant

Median + 95% CI is given.

Significant as compared to antibody reactivity after first vaccination: * p < 0.05; ** p < 0.01; *** p < 0.001; **** p < 0.0001

**Figure S6**

**Figure S6** **Correlation between time after alloHCT (in months) and IgG-anti-spike-1 antibody production in patients with hematological disorders in relation to the number of vaccinations;** A) after 1^st^ vaccination, B) after 2^nd^ vaccination, C) after 3^rd^ vaccination.

Individual values are given. R = correlation coefficient (Spearman’s rank) p = significance level. AU: arbitrary units

---- = normal range

**Figure S7**

**Figure S7** **Correlation between anti-spike-1 IgG antibody reactivity and laboratory parameters in alloHCT patients after the first (A), second (B) and third (C) vaccination**. Individual values are given. r = correlation coefficient (Spearman’s rank); p = significance level; s = slope; AU: arbitrary units

**Figure S8**


**Figure S8** **Effect of therapeutic or prophylactic treatment with monoclonal anti-SARS-CoV-2 an­ti­bodies on antibody reactivity in 16 patients with hematological disorders after alloHCT (a-p).**

Dates for antibody testing, application of antibodies (Evusheld^®^, Xevudy^®^, Ronapreve^®^), vac­cination (B=BNT162b2, M=mRNA-1273), and COVID-19 infection are indicated.

AU: arbitrary units

⚫ = IgG antibodies to spike-1 ◆ = IgG antibodies to spike-RBD ◼ = IgG antibodies to nu­cleocapsid
